# Detection of pulmonary tuberculosis among patients with cough attending outpatient departments in Dar Es Salaam, Tanzania: does duration of cough matter?

**DOI:** 10.1186/1472-6963-9-112

**Published:** 2009-07-01

**Authors:** Esther S Ngadaya, Godfrey S Mfinanga, Eliud R Wandwalo, Odd Morkve

**Affiliations:** 1 Centre for International Health, University of Bergen, Bergen, Norway; 2 Muhimbili Medical Research Centre, National Institute for Medical Research, Dar es Salaam, Tanzania; 3 Ministry of Health and Social Welfare, National Tuberculosis and Leprosy Control Programme, (NTLP), PO Box 9083 Dar es Salaam, Tanzania; 4 Management Sciences for Health, Dar es Salaam, Tanzania

## Abstract

**Background:**

According to WHO estimates, tuberculosis case detection rate in Tanzania is less than 50% and this poses a major challenge to control tuberculosis in the country. Currently, one of the defining criteria for suspecting tuberculosis is cough for two weeks or more. We wanted to find out whether the prevalence of tuberculosis was different in patients who reported cough for two weeks or more, compared to patients with cough for less than two weeks.

**Methods:**

We conducted a cross sectional study in six health facilities in Dar es Salaam, between September and October 2007. All patients aged five years and above with cough were screened for pulmonary tuberculosis (PTB) by smear microscopy. Patients were divided into two groups, those who coughed for less than two weeks (<2 wks) and those who coughed for two weeks or more (≥ 2 wks).

**Results:**

A total of 65,530 patients attended outpatients department (OPD). Out of these, 2274 (3.5%) patients reported cough. Among patients who reported cough, 2214 (97.4%) remembered their cough duration. One thousand nine hundred and seventy three patients (89.1%) coughed for ≥ 2 wks as compared to 241 (10.9%) patients who coughed for <2 wks. Of those who coughed for two weeks or more, 250 (12.7%) had smear positive PTB, and of those who had coughed for less than two weeks, 21 (8.7%) had smear positive PTB. There was no statistically significant difference in prevalence of smear positive tuberculosis among the two groups (Pearson Chi-Square 3.2; p = 0.074).

**Conclusion:**

Detection of smear positive PTB among patients who coughed for less than two weeks was as high as for those who coughed for two weeks or more.

## Background

It is more than a decade since tuberculosis (TB) was a global emergency disease, with a call for stronger international effort to fight it. Nevertheless, case detection is still low, particularly in the developing world, where this poses a major challenge [1]. Efforts made by most of the developed world in early case detection and prompt treatment constitute the basis for TB situation seen in these countries today [2]. While good TB treatment success rates has been achieved using directly observed short course therapy (DOTS), low case detection rates remain an obstacle to the long-term success of TB control programs in the developing world [3,4].

DOTS coverage in Tanzania is 100%, but case detection rates of new smear positive cases have shown a decreasing tendency from 53% in 1998 to 45% in 2005 [2]. Since active case finding has been found not to be cost effective in many studies [5,6], WHO recommends passive over active case finding [7]. Under passive case finding a patient is required to report to a health facility for care. In Tanzania, a patient to be recognized as a TB suspect needs to report to a health facility with cough for 2 or more weeks with or without accompanying symptoms. This depends to a large extent on patients' self initiative, socio-economic status and knowledge, and on degree of alertness of health workers [8]. According to WHO estimates, less than 50% of the smear positive TB cases are detected. Therefore, more effort is needed to increase case detection.

The objective of this study was therefore, to estimate prevalence of smear positive PTB among patients aged five years and above with a duration of cough of either less than two weeks or for two weeks or more, attending outpatient departments in Dar es Salaam.

## Methods

### Setting

We conducted the study in six health facilities in Dar es Salaam, which contain a quarter of all TB cases notified in the country [9]. Dar es Salaam is located in the eastern part of Tanzania, and is administratively divided into three districts namely Kinondoni, Temeke and Ilala with populations of 1,083,913, 768,451 and 634,924, respectively [10].

### Study design and data collection

This was a cross sectional hospital based study which was conducted in all three municipal hospitals of Dar es Salaam namely Mwananyamala, Amana and Temeke. Three government health centres were selected with equal probability from a list of all health centres obtained from each municipal. The selected health centres were Magomeni, Tabata and Zakiem. All patients aged five years and above, attending the outpatient clinics and reporting cough, regardless of the duration, were perceived as TB suspects and screened for TB by smear microscopy. Study clinicians from the same hospitals were trained. The clinicians registered all patients with cough in a study register and then requested the patients to submit three sputum samples as per national guidelines. Study registers also contained information on patients' socio-demographic characteristics, cough duration in days or weeks and sputum results.

Standard procedure for diagnosis of pulmonary tuberculosis in Tanzania is through passive case finding, where all patients with cough for two or more weeks should collect three sputum samples in the form of "spot-morning-spot" [11].

The study was conducted at a time when Central Tuberculosis Reference Laboratory (CTRL), the laboratory responsible for performing quality assurance, was conducting quality assurance using Lot Quality Assurance System (LQAS). In general, the results of all laboratories under the study were satisfactory. In addition, training was conducted to make sure that procedures for smear microscopy are harmonized. The quality check for the submitted samples was done according to routine National tuberculosis and leprosy Control Program (NTLP) guidelines [11].

Sample size calculation was done using Epi info version 6 with the assumptions that estimated prevalence of TB among patients with cough of two or more weeks was 0.63% [9] and prevalence of TB among patients with cough of less than two weeks would be half of that (0.32%). Using these assumptions, the minimum calculated sample size was 2125, we recruited 2274 TB suspects.

### Operational definitions

A PTB suspect: Any patient aged five years or more attending the outpatient department with cough, regardless of the duration.

A smear positive PTB patient is a patient who has at least two positive sputum samples for acid fast bacilli.

### Ethical considerations

The proposal was granted ethical clearance by the Tanzania Medical Research Coordinating Committee. Informed verbal consent was obtained from each interviewee before enrolment into the study. Patients with one smear positive sputum sample were referred to the district tuberculosis and leprosy coordinator (DTLC) for treatment and follow up using NTLP procedures. All patients with PTB were also referred to the DTLC for treatment. Non-TB patients were treated according to the diagnosis made.

### Analysis

Data collected was double entered, cleaned and coded using Epi-info version 6 (Centre for Diseased Control and Prevention, Atlanta, GA, USA). Analysis was done using SPSS version 14 for Windows (SPSS Inc, Chicago, IL, USA). The proportion of patients with smear positive PTB was calculated according to cough duration. Possible associations between PTB and patients' socio-demographic characteristics as well as cough duration were explored. Pearson chi-square and Wald statistic was used to compare group difference for categorical variables. Logistic regression was performed for age groups and occupation. Differences were considered statistically significant if p ≥ 5%. Nineteen (1.3%) patients had only one smear positive sputum sample and were excluded from the analysis.

## Results

### Baseline characteristics

During the study period, a total of 65,530 patients attended outpatients department (OPD). Out of these, 2274 (3.5%) patients reported cough. Among patients who reported cough, 2214/2274 (97.4%) remembered their cough duration and submitted all three sputum samples. In the analysis, the total number of patients thus is 2214. Due to missing data for some variables, the denominator for percentages given varies from 2197 to 2214. Sex distribution among the study population was almost equal with 1115 females (50.3%). The majority (1241/2197, 56.4%) were between 18–35 years, only 137/2197 (6.2%) were below 18 years. More than half of the patients (1378/2203, 62.6%) were couples (married and cohabiting). 1880/2198 (85.5%) had standard seven education. Most of the study population (1155/2202, 52.6%) were self employed and the rest were either employed (8.5%), students (7.6%) or peasant (4.9%). More than 26% were economically inactive. There was no statistically significant difference in socio-demographic characteristics between patients who had coughed for less than two weeks and those who had coughed for two weeks or more. The results of cross tabulation for binary variables and logistic regression for multi nominal variables for age groups and occupation did not show any statistically significant difference.

### Comparison of sputum positive cases by cough duration

**Figure 1:**
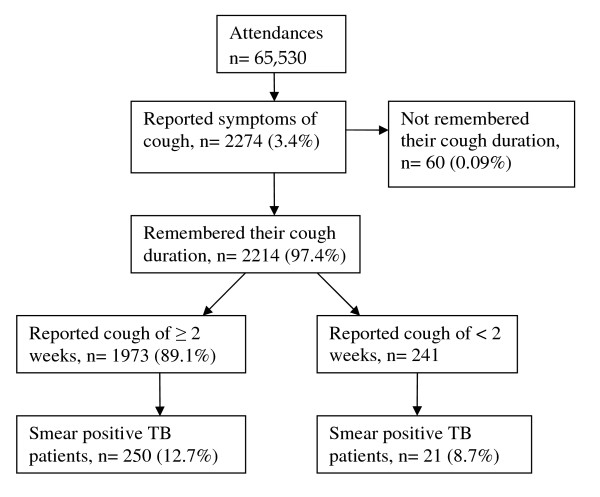
**Sputum positive cases by cough duration**.

As summarized in figure 1, 1973 (89.1%) patients had coughed for two or more weeks as compared to 241 (10.9%) who had coughed for less than two weeks. Of those who coughed for two weeks or more, 250 (12.7%) had smear positive PTB, and of those who had coughed for less than two weeks, 21 (8.7%) had smear positive PTB. There was no statistically significant difference among smear positives patients between the two groups (Pearson Chi-Square 3.2; p = 0.074).

### Comparison of smear positive PTB patients with cough duration according to gender and diagnostic centre

As shown in table 1, 163 (n = 271, 60.1%) males and 108 (n = 271, 39.9%) females were smear positive. Males were more likely to be smear positive in both groups as compared to females (X^2 ^= 90.1; p = 0.001).

**Table 1:** Smear positive patients: Gender comparison of cough in less or more than two weeks.

**Positive smears**	**Cough duration**		**Total**
		
	**Two weeks or more**	**Less than two weeks**	
Overall*	250/1955 (12.8%)	21/240 (8.7%)	271/2195 (12.3%)

Males**	149/250 (59.6%)	14/21 (66.7%)	163/271 (60.1%)

Females**	101/250 (40.4%)	7/21 (33.3%)	108/271 (39.9%)

• *Pearson Chi-Square 3.2; df 1; p = 0.073

• **Pearson Chi-Square 90.1; df 1; p = 0.001

• Some totals do not add up to 2214 due to missing information for some variables.

Table 2, shows the proportions of new adult outpatients who attended municipal hospitals and those who attended health centres by cough duration. One thousand four hundred and fifty one out of 1973 (73.5%,) patients who attended municipal hospitals, had coughed for two or more weeks as compared to 522 out of 1973 (26.4%) who attended health centres. The difference was statistically significant at X^2 ^= 38.8 and p = 0.001.

**Table 2:** Comparison of smear positive TB patients with cough duration according to diagnostic centre

**Cough duration**					
	**Two weeks or more** **N = 1973**		**Less than two weeks** **N = 241**		
			
**Area of diagnosis**	**Patients with cough**	**Proportion of smear +ve patients among coughers % (n/N)**	**Proportion of Patients with cough**	**Proportion of smear +ve patients among coughers % (n/N)**	**P values **^¥^

*Municipal Hospitals*					

Mwananyamala	682	14.8 (101/682)	65	10.8 (7/65)	0.376
Amana	312	21.2 (66/312)	27	11.1 (3/27)	0.214
Temeke	457	9.4 (43/457)	39	17.9 (7/39)	0.155
*Hospitals Total	**1451**	**14.5 (210/1451)**	**131**	**13 (17/131)**	0.640

*Health Centres*					

Magomeni	207	10.1 (21/207)	72	5.6 (4/72)	0.240
Tabata	80	1.3 (1/80)	15	0	
Zakiem	235	7.7 (18/235)	23	0	
*Health Centres Total	**522**	**7.7 (40/522)**	**110**	**3.6 (4/110)**	0.132

• *Pearson Chi-Square = 38.8; p = 0.001.

## Discussion

The main finding of this study is that detection of smear positive PTB among patients attending OPD in Dar es Salaam who had coughed for less than two weeks was as high as for those who had coughed for two weeks or more. Therefore, it may not be necessary for someone to cough for two weeks before *mycobacterium tuberculosis *becomes evident in the sputum. This finding has implication to the NTLP strategies on TB/HIV control activities and service delivery, since it raises the question whether the two weeks duration of cough should be kept as part of the definition of a TB suspect.

According to existing NTLP guidelines, a patient is not suspected to have tuberculosis unless the patient has been coughing for two or more weeks, with exception of those who are coughing blood. In some countries, a limit of three weeks is widely used. The NTLP, uses passive TB case finding depending largely on patients reporting to the OPD of health facilities. Our study indicates that pulmonary tuberculosis among OPD attendees with a history of cough is not significantly less frequent, when duration of cough is shorter than two weeks. Whether this also applies to other high prevalence settings needs to be confirmed by further studies. Using the current criteria, we might be missing quite a big number of TB cases, since those who report at the out patient department with cough of less that two weeks are not screened for TB under routine program. However, more studies are needed to assess the cost effectiveness of the screening approach that we have used here.

Taking into consideration the still increasing prevalence of TB/HIV co-infection, it may pay off to screen for TB regardless of cough duration, if we really intend to eradicate TB by year 2050 [2]. A study conducted in Brazil, a country with prevalence of HIV/AIDS in the adult population of less than 1% and annual incidence of smear positive TB patients/100,000 population of 26, reported that the probability of detecting TB case does not depend on the duration of cough [12]. Our study in Tanzania, where prevalence of HIV/AIDS is 7% and annual incidence of smear positive TB patients/100,000 population is 147, support this finding [11].

Screening, regardless of cough duration will accelerate early TB case detection and treatment. This is critically important especially for individuals co-infected with HIV/AIDS, as it will reduce morbidity and mortality. In addition, it could shorten the duration of TB transmission, as it might reduce diagnostic delays, since whoever is coughing will be immediately investigated for TB.

Apparently, the screening approach might increase laboratory workload. However, considering our study experience, where we had 241 extra TB suspects who coughed for less than two weeks, and assuming that each patient submits three samples, it will add up to 723 smear microscopy slides from all six health facilities in one month. Extrapolating this to 20 working days in a month, there will be an increase of about 6 smear microscopy slides per facility per day. Therefore, the increased workload appears to be manageable.

Our finding that more male than female patients were detected is consistent with other studies [2]. The reason for this is not known, but one study has shown that women have inability to produce good and quality sputum [13]. Nevertheless, this indicates that expanding TB case detection activities to maternal and child health clinics, antenatal and family planning clinics may lead to detection of more females suspects.

Detection of smear positive TB in patients who reported to have been coughing for less than two weeks could probably be explained (though not investigated in this study) partly by patient's ignorance and their perspectives on duration of illness. In addition, such patients could have been in several other health facilities that could have suspected TB if medical personnel had good clinical suspicion index for TB. Health systems have been found to play a substantial part in TB diagnosis delay [14,15]. However, health seeking behaviour of the study participants before they present at the outpatient departments was not investigated.

Private facilities were not sampled in our study, but in most cases, TB diagnostic services in Tanzania are through government facilities, as the service is free of charge. Another limitation is that dispensaries were not included. Recall bias might also play some part for over- or underestimating the duration of illness as well as not reporting cough symptom.

## Conclusion

Detection of smear positive PTB among patients who had coughed for less than two weeks was as high as for those who had coughed for more than two weeks among patients attending outpatient departments in Dar es Salaam. This question the appropriateness of defining a certain duration of cough as a prerequisite for being recognized as a TB suspect

## Competing interests

The authors declare that they have no competing interests.

## Authors' contributions

ESN is the primary author who was responsible for conceiving the research idea, designing the study, collection of data, analysis and interpretation of the results and writing of the draft and final manuscript. She is also the corresponding author. GSM, ERW and OM participated in proposal write up, data analysis and interpretation of the results, and writing of the draft and final manuscript.

## Pre-publication history

The pre-publication history for this paper can be accessed here:


